# Biodegradable, Thermally Stable, and Programmable Cellulosic Bioplastics Enabled by Supramolecular Stimulated Mediation

**DOI:** 10.34133/research.1098

**Published:** 2026-02-05

**Authors:** Junjie Zhou, Geyuan Jiang, Minxin Wang, Lisha Sun, Haipeng Yu, Dawei Zhao

**Affiliations:** ^1^ Key Laboratory on Resources Chemicals and Materials of Ministry of Education, Shenyang University of Chemical Technology, Shenyang 110142, P. R. China.; ^2^ Shengjing Hospital of China Medical University, Shenyang 110000, P. R. China.; ^3^ State Key Laboratory of Woody Oil Resources Utilization, Key Laboratory of Bio-based Material Science and Technology of Ministry of Education, Northeast Forestry University, Harbin 150040, P. R. China.

## Abstract

Bioplastics derived from renewable food crops or agricultural feedstocks are alternatives to petrochemical materials, but it is challenging to balance their mechanical properties, thermal stability, and shapeability. Here, we report a thermally stimulated supramolecular bioplastic that employs polyethylene glycol to optimize the assembly of cellulose and polyvinyl alcohol molecules. The resulting bioplastic showed a reinforced supramolecular architecture, with a mechanical elastic modulus of 3.23 GPa and an impact resistance higher than 8.15 kJ·m^−1^. It also showed thermal stability from −40 to 135 °C while maintaining its structural integrity and toughness, giving it potential applications for various shaping processes, including weaving, pouring, and molding. The bioplastic could also undergo natural soil biodegradation within 55 d and exhibited promising recyclability and economic feasibility. This study provides a strategy for configuring supramolecular structures and enhancing the design and manufacture of bioplastics with optimal comprehensive properties.

## Introduction

**Fig. 1.**
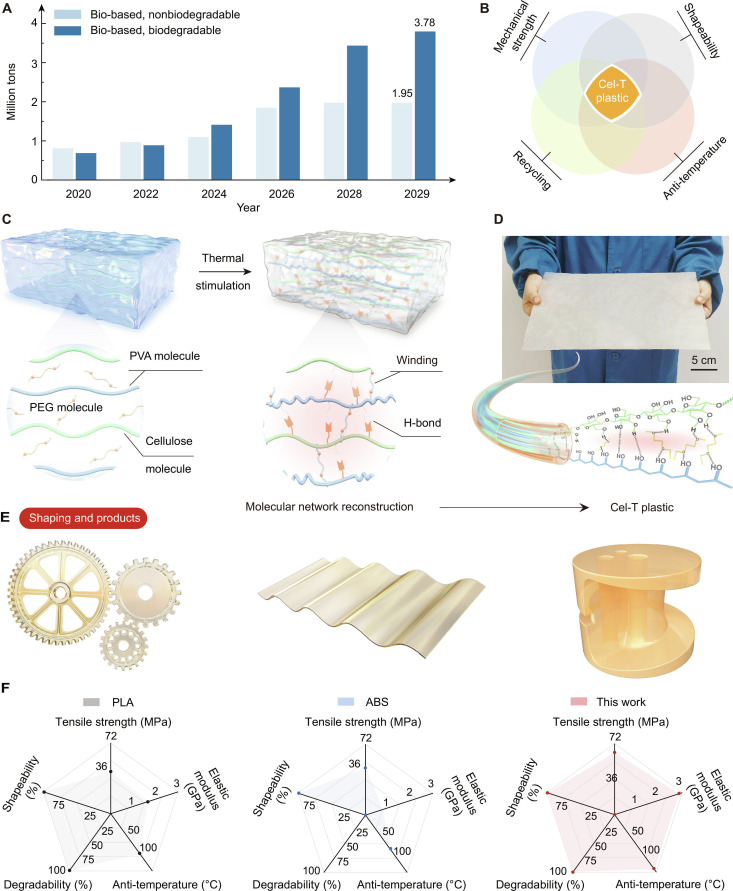
Conceptual scheme of the fabrication of Cel-T plastic through the design of thermally stimulated supramolecular architecture. (A) Bar chart of global production capacity and forecast of biodegradable and nonbiodegradable bioplastics from 2020 to 2029. (B) The concept intersection diagram of Cel-T plastic with mechanical properties, formability, thermal stability, and recyclability simultaneously. (C) Schematic diagram of thermal-stimulation-directed polyethylene glycol (PEG) modulation of densely ordered architectures within cellulose–polyvinyl alcohol (Cel–PVA) molecular networks. (D) Demonstration of the large-scale preparation of Cel-T plastic and schematic diagram of internal molecular interconnections. (E) Shaping and products of Cel-T plastic. (F) Radar plots comparing the key attributes of Cel-T plastic. PLA, polylactic acid; ABS, acrylonitrile-butadiene-styrene.

Due to the severe impact of nonbiodegradable petrochemical plastics on both the environment and human health [1–4] and increasing awareness of environmental protection, bioplastics have emerged as a popular alternative [5–9]. The global bioplastic production capacity has been steadily increasing since 2020, with projections suggesting that it will reach 5.73 million tons by 2029 (Fig. 1A) [10–12]. Due to this sustained growth, renewable and biodegradable cellulose materials have become promising alternatives to conventional plastics [13–16].

Bioplastics are either bio-based or biodegradable materials and include polylactic acid (PLA), biomass-based plastics, and polyalcohol ester plastics [17–21]. However, their mechanical properties, high-temperature resistance, and plastic shapeability lag behind those of petrochemical plastics. The mechanical properties and thermal stability of bioplastics can be enhanced by designing stimuli-responsive supramolecular networks [22–26]. However, there is a notable gap in the plastic injection molding and processing behaviors of bio-based plastics compared with those of their petrochemical counterparts [27,28].

Here, we present a supramolecular cellulosic bioplastic, termed Cel-T plastic, which simultaneously exhibits mechanical robustness, thermal stability, controllable shapeability, and recyclability (Fig. 1B). These attributes were achieved by incorporating thermally stimulated polyethylene glycol (PEG) molecules, which enhanced the supramolecular architecture of cellulose and polyvinyl alcohol (PVA). Within Cel-T plastic, PEG functioned as a cross-linker via hydrogen bonds (H-bonds), which facilitated PVA recrystallization and formed a denser and more resilient supramolecular network (Fig. 1C).

The scalable Cel-T plastic (Fig. 1D) demonstrated a tensile strength exceeding 63 MPa and an elastic modulus surpassing 3 GPa, as well as remarkable thermal stability and toughness from −40 to 135 °C. Cel-T plastic also showed 3-dimensional (3D) shaping capabilities in injection molding, compression molding, and continuous manufacturing (Fig. 1E) comparable to those of PLA and acrylonitrile-butadiene-styrene (ABS). Furthermore, Cel-T plastic displayed an attractive impact resistance and was completely biodegraded in soil (Fig. 1F). This study presents an efficient approach to designing bioplastics with improved mechanical, thermal, and shaping properties that may reduce petrochemical plastic pollution.

## Results and Discussion

### Fabrication and shaping of Cel-T plastic

Cellulose was dissolved in the ionic liquid 1-butyl-3-methylimidazolium chloride ([Bmim]Cl) to create a homogeneous system of cellulose macromolecules [29–35]. PVA was then incorporated, which formed a cellulose–PVA (Cel–PVA) supramolecular network. [Bmim]Cl was replaced with deionized water, which formed a Cel–PVA hydrogel (Fig. S1). This facilitated the recovery and recycling of the [Bmim]Cl via rotary evaporation. The resulting hydrogel was treated with PEG to form the Cel–PVA–PEG system. Finally, thermal stimulation at 80 °C for 6 h induced the PEG-mediated reorganization of the Cel–PVA supramolecular network, which yielded an extensible Cel-T plastic with a cellulose content of 54 wt% (Fig. 1D and Fig. S2).

Cel-T plastic contained abundant polar hydroxyl (–OH) groups from cellulose and PVA molecules, along with ether bonds (–O–) and terminal –OH groups in the PEG molecular chain. As a result, intermolecular interactions were primarily characterized by electrostatic interactions and H-bonds. Molecular dynamics (MD) simulations were conducted on 3 distinct systems: the Cel–PVA hydrogel (Fig. S3), the Cel–PVA–PEG system after the introduction of PEG, and the Cel-T plastic state after thermal stimulation.

**Fig. 2.**
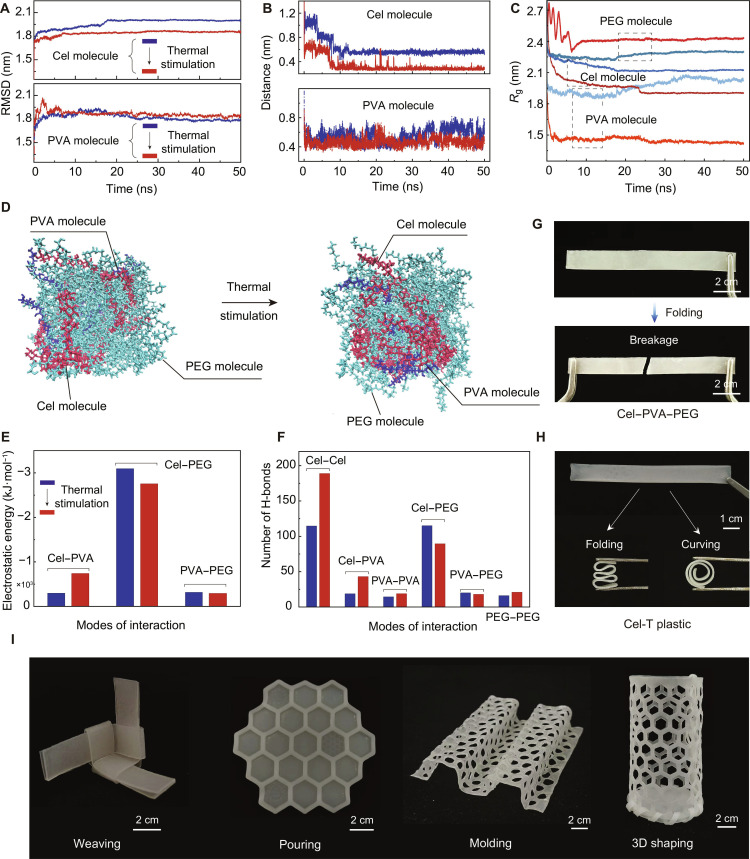
Molecular dynamics (MD) simulations and various shapings of Cel-T plastic. (A) Root-mean-square deviation (RMSD) of cellulose and PVA molecules in the Cel–PVA–PEG system and Cel-T plastic simulated by MD. (B) End-to-end distance of cellulose and PVA molecules in the Cel–PVA–PEG system and Cel-T plastic. (C) Radius of gyration (*R*_g_) of cellulose, PVA, and PEG molecules in the Cel–PVA–PEG system and Cel-T plastic. (D) MD snapshots of the Cel–PVA–PEG system and Cel-T plastic. (E) Comparison of the electrostatic energy between the Cel–PVA–PEG system and Cel-T plastic. (F) Number of H-bonds in the Cel–PVA–PEG system and Cel-T plastic. (G) The optical image of the Cel–PVA–PEG system shows that brittle fracture occurred under the influence of bending. (H) Optical images of Cel-T plastic demonstrate excellent toughness and foldability. (I) Cel-T plastic exhibits appealing capabilities in weaving, pouring, molding, and 3-dimensional (3D) shaping.

Figure 2A shows that the root-mean-square deviation (RMSD) of the cellulose and PVA molecules indicated minimal changes during thermal stimulation, suggesting that Cel–PVA maintained a stable, rigid framework. To further investigate changes in the supramolecular architecture of Cel-T plastic, we compared the end-to-end distance (Fig. 2B) and the radius of gyration (*R*_g_, Fig. 2C) of cellulose and PVA molecules before and after thermal stimulation. In contrast to the Cel–PVA–PEG system, both the *R*_g_ values and end-to-end distances of cellulose and PVA molecules in Cel-T plastic were lower, particularly the *R*_g_ of PVA molecules. This reduction suggests that thermal stimulation enhanced entanglements between cellulose and PVA, as well as the crystalline arrangement of PVA, which formed a more densely knit supramolecular network (Fig. 2D).

To examine the role of PEG as the cross-linking agent within Cel-T plastic, we assessed the effects of thermal stimulation on the supramolecular network. The increase in *R*_g_ (Fig. 2C) and RMSD (Fig. S4a) of PEG suggested that thermal stimulation enhanced their chain mobility. This caused them to expand and become uniformly distributed within the Cel–PVA supramolecular network. Thermal stimulation also decreased the electrostatic energy and the number of H-bonds between cellulose and PEG, as well as between PVA and PEG molecules (Fig. 2E and F and Fig. S4b). This indicates that excess PEG molecules did not promote the densification of the Cel-T plastic supramolecular network and detached from the material.

Compared with the Cel–PVA–PEG system, the electrostatic energy of Cel–PVA (Fig. 2E) and the number of H-bonds between Cel–Cel, Cel–PVA, and PVA–PVA in Cel-T plastic were significantly higher. This confirmed the reconstruction and densification of the supramolecular network within Cel-T plastic. This molecular-scale enhancement imparted mechanical strength to Cel-T plastic, allowing it to maintain structural integrity without being damaged, even during large-scale bending and folding (Fig. 2G and H and Fig. S5). As shown in Fig. S6, Cel-T plastic supported a weight approximately 6,250 times its own weight without being damaged.

This supramolecular architecture mediated by PEG molecules may give Cel-T plastic a range of 3D shaping behaviors in weaving, injection molding, compression molding, and other processes that may rival those of commercial plastics (Fig. 2I and Fig. S7). The molded Cel-T plastic also demonstrated remarkable mechanical toughness by supporting a load of 5 kg while maintaining its structural integrity, despite weighing only 0.7 g (Fig. S8).

### Microstructural evolution of Cel-T plastic

**Fig. 3.**
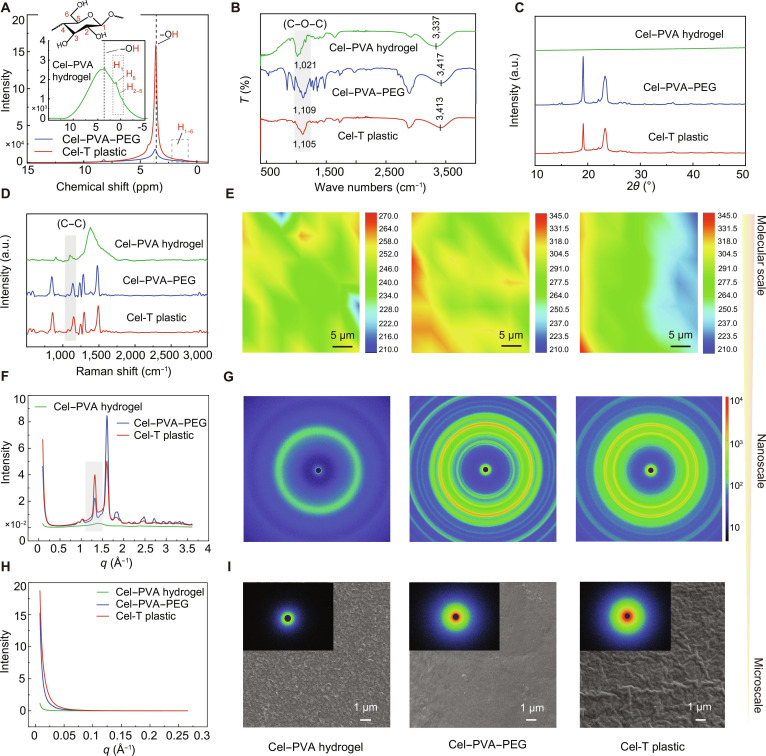
Microscopic characterizations and structures of Cel-T plastic. (A to E) Molecular-scale characterization of the Cel–PVA hydrogel, the Cel–PVA–PEG system, and Cel-T plastic represented by solid-state ^1^H nuclear magnetic resonance (NMR) patterns (A), infrared (IR) patterns (B), x-ray diffraction (XRD) patterns (C), Raman patterns (D), and 2-dimensional (2D) Raman images (E). (F and G) Nanoscale characterization of the Cel–PVA hydrogel, the Cel–PVA–PEG system, and Cel-T plastic represented by wide-angle x-ray scattering (WAXS) patterns (F) and 2D WAXS images (G). (H and I) Microscale characterization of the Cel–PVA hydrogel, the Cel–PVA–PEG system, and Cel-T plastic represented by small-angle x-ray scattering (SAXS) patterns (H) and 2D SAXS and scanning electron microscopy (SEM) images (I).

The microstructural evolution of Cel-T plastic was investigated through a combination of molecular-scale, nanoscale, and microscale characterization techniques. The molecular structure of the supramolecular networks of Cel-T plastic was analyzed using solid-state ^1^H nuclear magnetic resonance spectroscopy (Fig. 3A). After adding PEG to the Cel–PVA supramolecular network, the –OH peak of cellulose molecules became sharper and more pronounced. This indicated enhanced molecular cross-linking via H-bonding within the Cel–PVA–PEG system. Subsequent thermal stimulation caused the –OH peak to shift from 3.678 to 3.629 ppm and undergo an increase in intensity. The shift suggested a reorganization of the supramolecular network in Cel-T plastic toward a denser and more compact architecture. This is consistent with the findings of MD simulations and Fourier transform infrared spectroscopy (Fig. 3B). Specifically, the O–H stretching band shifted from 3,337 to 3,413 cm^−1^ after thermal stimulation, indicating an enhancement of hydrogen bonding within Cel-T plastic.

X-ray diffraction and Raman spectroscopy analyses revealed that Cel-T plastic exhibited a compact supramolecular configuration (Fig. 3C). The peak at 1,140 cm^−1^ became sharper and more intense after thermal stimulation (Fig. 3D) because the crystallization behavior of the PVA molecular chain was facilitated and enhanced by the inclusion of PEG (Fig. S9a to d). The 2-dimensional (2D) Raman images of Cel-T plastic showed a uniform color distribution (Fig. 3E), indicating a dense and uniform structure.

The wide-angle x-ray scattering (WAXS) curves, 2D WAXS images, and small-angle x-ray scattering (SAXS) data of the Cel–PVA hydrogel, the Cel–PVA–PEG system, and Cel-T plastic are shown in Fig. 3F to H. A comparison of the data showed that Cel-T plastic showed sharper peaks (Fig. 3F and H) and more pronounced 2D WAXS (Fig. 3G). This was attributed to synergistic interactions between the 3 molecular components. The cellulose macromolecules served as the molecular framework and established the architecture of Cel-T plastic. The locally crystallized PVA molecules acted as the reinforcing phase, thereby increasing the structural strength of Cel-T plastic. PEG acted as a cross-linking agent and enhanced the structural toughness via hydrogen-bonding interactions with both cellulose and PVA.

In the scanning electron microscopy (Fig. 3I and Fig. S9e and f) and 2D SAXS images (upper left corner of Fig. 3I), Cel-T plastic exhibited a more compact and flat micromorphology compared to the Cel–PVA–PEG system. This supramolecular design is expected to endow Cel-T plastic with robust mechanical properties and weathering resistance.

### Mechanical properties

**Fig. 4.**
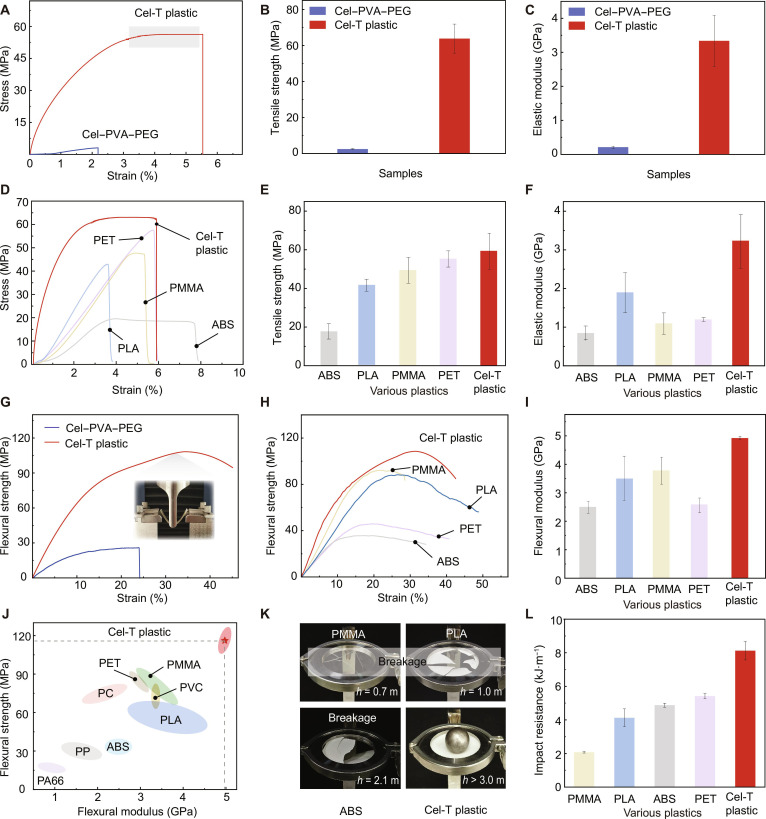
Mechanical performance of Cel-T plastic. (A to C) Comparisons of tensile properties between the Cel–PVA–PEG system and Cel-T plastic through tensile stress–strain curves (A) and bar charts of the tensile strength (B) and the elastic modulus (C). (D to F) Comparisons of the tensile properties of Cel-T plastic with those of PLA, polymethyl methacrylate (PMMA), ABS, and polyethylene terephthalate (PET) through tensile stress–strain curves (D) and bar charts of the tensile strength (E) and the elastic modulus (F). (G) Flexural stress–strain curves of Cel–PVA–PEG system and Cel-T plastic. (H and I) Comparisons of the flexural properties of Cel-T plastic with those of PLA, PMMA, ABS, and PET through flexural stress–strain curves (H) and bar charts of the flexural strength (I). (J) Comparison of tensile strength and elastic modulus between Cel-T plastic and commercial plastics. (K and L) Cel-T plastic’s robustness through digital photographs of the materials (K) and impact resistance (L) during free-fall impact performance tests, compared with those of commercial plastics. PA66, polyamide 66; PP, polypropylene; PC, polycarbonate; PVC, polyvinyl chloride.

We comprehensively analyzed the mechanical properties of Cel-T plastic and compared them to those of the Cel–PVA–PEG system, PLA bioplastic, and various petrochemical plastics, including polymethyl methacrylate (PMMA), ABS, and polyethylene terephthalate (PET). As illustrated in Fig. 4A, under a stress exceeding 50 MPa, the strain of Cel-T plastic did not surpass 3%, indicating high rigidity. When subjected to a high and stable tensile stress, Cel-T plastic underwent plastic deformation, with an ultimate tensile strength of 63.86 ± 8.07 MPa. This value surpassed that of the Cel–PVA–PEG system, which was only 2.59 ± 0.38 MPa (Fig. 4B). The elastic modulus of Cel-T plastic was 3.3 ± 0.7 GPa, which was more than 15 times greater than that of the Cel–PVA–PEG system, which was 0.211 ± 0.029 GPa (Fig. 4C). The thermally stimulated enhancement of PEG chain mobility facilitates the rearrangement and strengthening of the Cel–PVA H-bond network. This optimization of crystalline domains directly contributes to the improved macroscopic mechanical properties. Cel-T plastic demonstrated a tensile strength and an elastic modulus superior to those of ABS, PLA, PMMA, and PET (Fig. 4D to F and Fig. S10). The mechanical toughness of Cel-T plastic reached 3.12 MJ·m^−3^, which exceeds those of ABS, PLA, PMMA, PET, and several other previously reported plastics (Fig. S11).

Cel-T plastic also exhibited resistance to deformation during the bending test. Its dense, ordered structure and crystalline domains facilitated stress dispersion, which enhanced both its toughness and energy absorption capacity. This distinct supramolecular architecture gave Cel-T plastic a high flexural strength and modulus that surpassed those of other reported bioplastics [36–39]. Compared with the Cel–PVA–PEG system, ABS, PLA, PMMA, PET, polyvinyl chloride (PVC), polyamide 66 (PA66), and other plastics (Fig. 4G to J and Fig. S12), Cel-T plastic exhibited higher flexural strength and flexural modulus of 108.6 MPa and 4.9 GPa, respectively. Due to its mechanical properties, Cel-T plastic may make a promising alternative to petrochemical plastics in applications requiring high specific tensile and bending strength.

Cel-T plastic also showed an impact resistance superior to those of other plastics. During the free-fall impact test, a 256-g iron ball dropped from different heights severely damaged PLA, PMMA, and ABS at lower heights. In contrast, Cel-T plastic retained its structural integrity even when the ball was dropped from higher than 3 m (Fig. 4K). The impact resistance of Cel-T plastic was measured as 8.15 ± 0.55 kJ·m^−1^, surpassing those of PMMA, PLA, ABS, and PET (Fig. 4L). As illustrated in Fig. S13, Cel-T plastic also exhibited exceptional puncture resistance. In tests involving an 80-g steel needle dropped from heights of less than 1.5 m, PLA, PMMA, ABS, and PET all suffered breakage and damage. In contrast, Cel-T plastic merely showed indentations of varying depths while maintaining its overall structural integrity.

### High/low-temperature stability

**Fig. 5.**
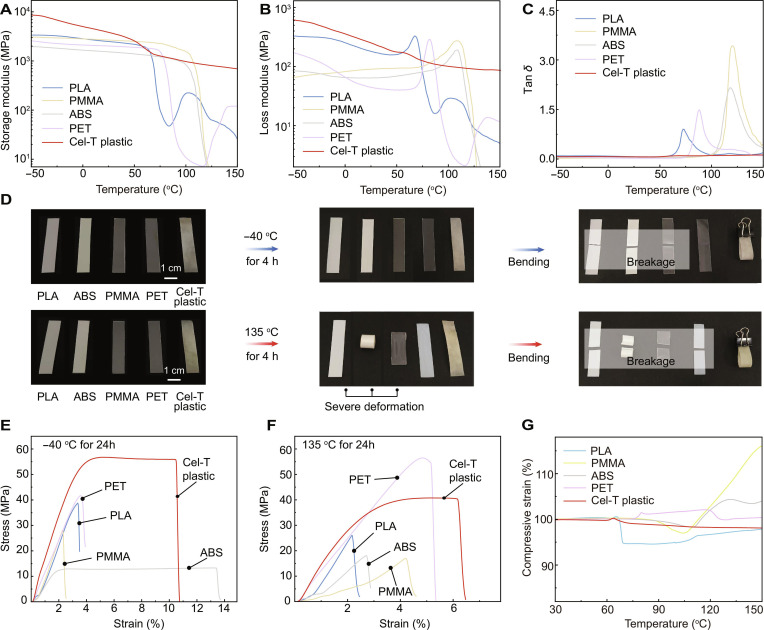
Excellent structural stability of Cel-T plastic at both low and high temperatures. (A to C) Dynamic mechanical analysis (DMA) test curves of Cel-T plastic, PLA, PMMA, ABS, and PET: storage modulus (A), loss modulus (B), and tan *δ* (C). (D) Anti-low-temperature and anti-high-temperature photos of Cel-T plastic, PLA, PMMA, ABS, and PET. (E) The tensile stress–strain curves of various plastics after exposed at −40 °C for 24 h. (F) The tensile stress–strain curves of various plastics after exposure to 135 °C for 24 h. (G) The compressive strain–temperature curves of plastics obtained by thermomechanical analysis (TMA) tests.

We also compared the thermal stability and low-temperature resistance of Cel-T plastic with those of PLA, PMMA, ABS, and PET. Dynamic mechanical analysis (Fig. 5A to C) revealed that Cel-T plastic displayed smooth curves for its storage modulus, loss modulus, and loss tangent (tan *δ*) from −40 to 135 °C. This indicated that its supramolecular architecture was stable across both high and low temperatures without deformation or softening, and this performance surpassed those of several previously reported bio-based plastics [40–42]. In temperature resistance tests, Cel-T plastic maintained its mechanical toughness and bendability after exposure to −40 and 135 °C for 4 h, respectively. In contrast, other plastics tested, including PLA, PMMA, ABS, and PET, became brittle, prone to breakage, or exhibited softening and deformation (Fig. 5D).

Subsequently, we investigated the mechanical properties of Cel-T plastic after prolonged exposure to both low and high temperatures. As shown in Fig. 5E, Cel-T plastic exposed to −40 °C for 24 h exhibited a tensile strength exceeding 55 MPa, which was superior to those of PLA, PMMA, ABS, and PET plastics. Even after being heated to 135 °C for 24 h, Cel-T plastic maintained a tensile strength of over 40 MPa, surpassing those of PLA, PMMA, and ABS plastics (Fig. 5F). These findings indicate that Cel-T plastic was suitable for applications requiring a high mechanical strength at high temperatures. Compared with PLA, PMMA, ABS, and PET plastics (Fig. 5G), Cel-T plastic presented negligible compressive strain recovery. It also showed thermal insulation properties, with a coefficient of thermal expansion of only 28.22 ppm·°C^−1^ (Fig. S14).

### Recyclability, biodegradability, biocompatibility, and economic feasibility

**Fig. 6.**
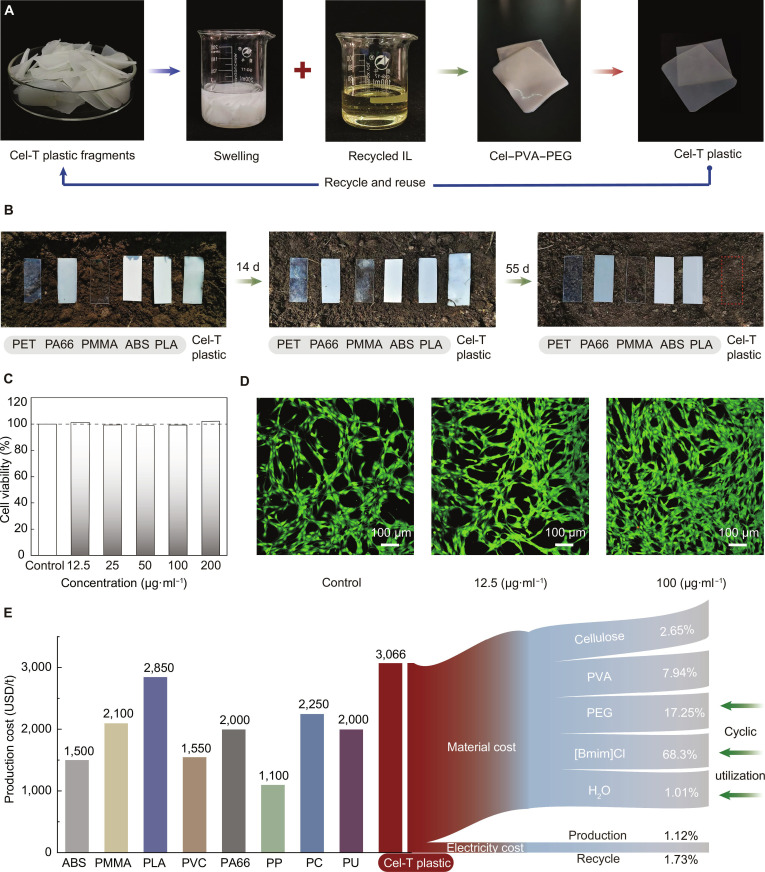
Recyclability, biodegradability, biocompatibility, and economic feasibility of Cel-T plastic. (A) Investigating the recyclability of Cel-T plastic. (B) The biodegradability tests of Cel-T plastic and commercial plastics under natural soil. (C) Investigating the cell viability of Cel-T plastic in different cell suspension concentrations. (D) Live staining confocal images of skin tissue cells co-cultured with different concentrations of Cel-T plastic for 24 h. (E) Comparison of the production costs of Cel-T plastic and commercial plastics, namely, ABS, PMMA, PLA, PVC, PA66, PP, PC, and polyurethane (PU). IL, ionic liquid; [Bmim]Cl, 1-butyl-3-methylimidazolium chloride.

Due to the inherent recyclability of cellulose and the reusability of [Bmim]Cl, our Cel-T plastic is expected to exhibit closed-loop recyclability. Figure 6A shows that Cel-T plastic fragments could be recycled through a straightforward process involving dissolution in [Bmim]Cl and subsequent reprocessing. The recycling procedure involved 2 steps. First, the fragments were swelled and dissolved in recycled [Bmim]Cl to recreate the Cel–PVA supramolecular system. Second, a PEG solution was subjected to thermal stimulation to produce the Cel-T plastic. The tensile strength of recycled Cel-T plastic remains above 85% of the initial value, and the elastic modulus retention rate exceeds 90% (Fig. S15). Meanwhile, the [Bmim]Cl recovery efficiency is about 85% in each cycle and the [Bmim]Cl residual content is negligible in the recycled plastic (Fig. S16). The recycled Cel-T plastic maintains excellent thermal stability and shapeability (Fig. S17).

The environmental sustainability was further enhanced by the degradability of Cel-T plastic. Within 55 d, Cel-T plastic underwent complete morphological disintegration and biodegradation (Fig. 6B). In contrast, conventional commercial plastics such as PET, PA66, PMMA, ABS, and PLA exhibited negligible degradation. The biodegradation of Cel-T plastic in natural soils can address concerns related to plastic pollution, positioning it as a viable alternative for managing commercial plastic waste by promoting both sustainability and resource circularity. Cel-T plastic also demonstrated biocompatibility, with cell survival rates exceeding 98.5% across various cell suspension concentrations. This is important for medical and food applications.

By exploring the solvent resistance of Cel-T plastic, we find that its mechanical strength and dimensional stability decrease under prolonged immersion in H_2_O, acidic, or alkaline solutions due to the hydrophilic nature of its components (Figs. S18a and b and S19). Moreover, by testing the long-term durability, Cel-T plastic exposed to 90% relative humidity at 35 °C for 7 d exhibited a tensile strength exceeding 50 MPa (Fig. S18c and d). Future work will focus on enhancing its solvent resistance through strategies such as mild surface modification or the introduction of hydrophobic moieties without compromising its biodegradability.

To illustrate the application potential of Cel-T plastic, we conducted a techno-economic analysis and compared Cel-T plastic with commercial plastics such as ABS, PMMA, PVC, PA66, polypropylene, PC, and polyurethane (Fig. 6E and Tables S1 and S2). The production costs of Cel-T plastic were approximately US$3,066 per ton. The primary cost drivers of Cel-T plastic are the availability of [Bmim]Cl and PEG, which accounted for 68.3% and 17.25% of total costs, respectively. Consequently, advancements in the recycling and reutilization of [Bmim]Cl and PEG could further reduce manufacturing costs and enhance the market competitiveness of Cel-T plastic. Electricity expenses represented only 2.85% of total expenses, underscoring this low-energy production process. Although Cel-T plastic had slightly higher production costs than petrochemical plastics, its exceptional mechanical properties, thermal stability, customizable formability, and reduced environmental impact may significantly enhance its competitiveness in the bioplastics market.

## Conclusion

This study presented Cel-T plastic, which addressed the trade-off between mechanical robustness, thermal stability, and 3D shaping capabilities. Cel-T plastic was characterized by a PEG-induced thermally stimulated supramolecular architecture, which imparted it with a tensile strength of 63.05 MPa, an elastic modulus of 3.23 GPa, a flexural strength of 108.6 MPa, and an impact resistance of 8.15 ± 0.55 kJ·m^−1^. It also demonstrated excellent operational stability across a wide temperature range of −40 to 135 °C. Compared with petrochemical plastics, the plant-based origins of Cel-T plastic significantly enhance its eco-friendliness, including its recyclability and biodegradability, which may help address the environmental concerns of traditional plastics. The economic feasibility of Cel-T plastic, with production costs of $3,066 per ton, makes it an attractive material for a broad spectrum of industrial applications. Its versatility for various shaping processes, including injection molding, extrusion, and 3D printing, may make it appealing to manufacturers. As sustainability becomes increasingly imperative to both brands and consumers, Cel-T plastic is a viable alternative to petrochemical plastics in the automotive, smart building, and healthcare industries.

## Materials and Methods

### Chemicals

Cellulose was extracted from poplar flour with an average polymerization degree of around 1,500, a crystallinity of 64%, and a diameter of 50 to 250 μm. PVA1788 was purchased from Bide Pharmatech (China), and PEG8000 was purchased from Aladdin (China).

### Preparation of the Cel–PVA supramolecular system and Cel–PVA hydrogel

According to a previous work, [Bmim]Cl was prepared by mechanically stirring 1-methylimidazole and 1-chlorobutane for 8 h at 85 °C. The 250-ml 3-mouth flask should be thoroughly washed and dried prior to synthesis. Then, dried cellulose and PVA were added to [Bmim]Cl at 85 °C for 6 h to physically dissolve the cellulose and PVA, resulting in a homogeneous Cel–PVA molecular system. A Cel–PVA colloid was obtained by placing the homogeneous system on a glass plate in a vacuum drying oven (85 °C and vacuum at −0.1 MPa) for 12 h. Finally, the Cel–PVA molecular system was obtained by placing the Cel–PVA colloid in water for 6 h.

### Preparation of the Cel–PVA–PEG system

The Cel–PVA hydrogel was soaked in PEG solution for 24 h at 65 °C to obtain the Cel–PVA–PEG system.

### Preparation of Cel-T plastic

The Cel–PVA–PEG system was placed in a vacuum oven at 80 °C for 6 h to obtain Cel-T plastic.

### [Bmim]Cl recycling

The mixture of [Bmim]Cl and water was collected after the fabrication of Cel–PVA hydrogel. This mixture followed by rotary evaporation to eliminate water and recover the recycled [Bmim]Cl.

### Mechanical properties

The tensile mechanical properties and bending stress–deflection behavior of the Cel–PVA–PEG system, Cel-T plastic, PLA, PMMA, ABS, and PET were measured using a UTM2503 electromechanical universal testing machine (Shenzhen SUNS Technology Stock CO., LTD.). The tensile speed of specimens was 10 mm·min^−1^ at room temperature. Bending stress–deflection testing was performed through the 3-point bending method (the span between the 2 support points was 40 mm). The specimens were tested at a constant speed of 5 mm·min^−1^ at room temperature.

For the free-fall impact and puncture resistance test, a 256-g iron ball was used as an impactor and an 80-g iron stick was used as an impactor, allowed to free-fall from a specific height. The drop height was adjusted until the sample was destroyed, and each material was tested at least 3 times. The energy absorbed during sample failure is defined as the loss of kinetic energy of the iron ball after impact, which is calculated using the following equation:

$$E=\frac{mv^{2}}{2d}$$ (1)

$$v=\sqrt{2\mathrm{gh}}$$ (2)

where *h* is the free-fall distance (m), *g* is the acceleration of gravity (9.8 m·s^−2^), *m* is the mass of the iron ball and stick, *d* is the thickness of the sample, and *ν* is the velocity of the iron ball or stick touching sample.

### Thermal property test

Dynamic mechanical analysis was performed on a TA Q800 analyzer (TA Instruments, USA). The samples were tested using tensile mode under an air atmosphere. The temperature ramp was from −50 to 150 °C at a frequency of 5 Hz and a heating rate of 5 °C·min^−1^. Thermomechanical analysis was performed on TMA/SDTA 2+ HT/1600/381 (TA instruments, USA). The samples were tested using the compress mode at a preloading force of 0.05 N within a range of 30 to 150 °C at a speed of 10 °C·min^−1^.

### Cytotoxicity test for human fibroblasts

The cytotoxicity of Cel-T plastic extract liquid was evaluated using the Cell Counting Kit-8 (CCK-8) assay. Extract liquids of Cel-T plastic at various concentrations (12.5, 25, 50, 100, and 200 μg/ml) were prepared by immersing them into complete media 24 h after sterilizing under ultraviolet irradiation. Normal human dermal fibroblast (NHDF) cells (4 × 10^3^ cells/well) were first seeded into 96-well plates and incubated with Dulbecco’s modified Eagle medium (DMEM) for 24 h (37 °C, 5% CO_2_). After removing the culture medium, the unattached NHDF cells were rinsed with phosphate-buffered saline (PBS) buffer solution. Then, extract liquids with diverse concentrations were incubated with NHDF cells for another 24 h. Finally, 10 μl of a CCK-8 solution was added to wells containing the cells and incubated together for 2 h. The cells treated without Cel-T plastic extract were applied as a blank control. The absorbance value at 450 nm was measured using an enzyme immunoassay analyzer.

The cell viability of the plastic is calculated as shown in Eq. (3):

$$\text{Cell viability}=\left[\frac{\mathrm{As}-\mathrm{Ab}}{\mathrm{Ac}-\mathrm{Ab}}\right]\times 100\%$$ (3)

Among them, *As* is the test hole absorbance, *Ac* is the control hole absorbance, and *Ab* is the white hole absorbance.

The cytotoxicity test was conducted using the live/dead staining to detect the viability changes of NHDF cells. Extract liquids of Cel-T plastic at various concentrations (10 and 200 μg/ml) were prepared by immersing them into complete media 24 h after sterilizing under ultraviolet irradiation. NHDF cells (2 × 10^4^ cells/well) were first seeded into a confocal dish and incubated with DMEM for 24 h (37 °C, 5% CO_2_). After removing the culture medium, the unattached NHDF cells were rinsed with PBS buffer solution. Then, extract liquids with diverse concentrations were incubated with NHDF cells for another 24 h. Before staining, the adherent cells were gently washed with PBS, the supernatant was removed, and the active esterase contained in the petri dish was removed. Enough solution (2 μM calcein-AM and 8 μM propidium iodide) was added, and the cells were incubated at room temperature for 30 to 45 min away from light. The dye work solution was sucked out to terminate incubation. The cells treated without Cel-T plastic extract were applied as a blank control. The stained cells were observed under a confocal microscope.

### Biodegradability test

To assess the biodegradability of various materials including PET, PA66, PMMA, ABS, PLA, and our Cel-T plastic, samples were prepared with dimensions of 50 × 30 × 1 mm and subsequently interred at a depth of 10 cm in natural soil. Periodically, these specimens were retrieved for evaluation, and their morphological alterations were recorded utilizing digital photography.

## Data Availability

The data that support the findings of this study are available from the corresponding authors upon reasonable request.

## Supplementary Materials

Supplementary 1 Notes S1 and S2 Figs. S1 to S19 Tables S1 and S2
