# Supplementary material for: Biodegradable, Thermally Stable, and Programmable Cellulosic Bioplastics Enabled by Supramolecular Stimulated Mediation
Source: Research (Wash D C). 2026 Feb 5;9:1098. doi: 10.34133/research.1098 (PMC12873063; doi:10.34133/research.1098)
Supplement: Supplementary 1 — Notes S1 and S2 Figs. S1 to S19 Tables S1 and S2 [file research.1098.f1.docx]

**Supplementary Materials**

**Biodegradable, Thermally Stable, and Programmable Cellulosic Bioplastics Enabled by Supramolecular Stimulated Mediation**

Junjie Zhou^1^, Geyuan Jiang^1^, Minxin Wang^1^, Lisha Sun^2*^, Haipeng Yu^3*^, Dawei Zhao^1,3*^

1. Key Laboratory on Resources Chemicals and Materials of Ministry of Education, Shenyang University of Chemical Technology, Shenyang 110142, P. R. China
2. Shengjing Hospital of China Medical University, Shenyang 110000, P. R. China
3. State Key Laboratory of Woody Oil Resources Utilization; Key Laboratory of Bio-based Material Science and Technology of Ministry of Education, Northeast Forestry University, Harbin 150040, P. R. China

* Corresponding to:

sunls@sj-hospital.org;

[yuhaipeng20000@nefu.edu.cn](mailto:yuhaipeng20000@nefu.edu.cn);

[daweizhao@syuct.edu.cn](mailto:daweizhao@syuct.edu.cn);

**Note S1. Characterization methods.**

The chemical structures of the Cel-PVA hydrogel, the Cel-PVA-PEG system and Cel-T plastic were subjected to solid-state nuclear magnetic resonance hydrogen spectroscopy (^1^H SNMR) analysis at 25 ℃ using a Bruker 600M (Bruker, Germany). The scanning electron microscopy (SEM) microstructure of samples was characterized by a JSM-IT800 microscope (JEOL, Tokyo, Japan) at an operating voltage of 10 kV. Fourier transform infrared (FTIR) spectra of samples were obtained by a Nicolet IS50 FTIR instrument (Thermo Fisher Scientific Inc., Waltham, MA, USA). Spectra of the Cel-PVA hydrogel and Cel-T plastic were measured in ATR mode, spectra of the Cel-PVA-PEG system was measured in KBr tablet press mode, with data recorded in the range of 500-4000 cm^-1^. The X-ray diffraction (XRD) pattern of sample was measured by a D/max 2200 X-ray diffractometer (Rigaku, Tokyo, Japan) α equipped with Ni-filtered Cu-K radiation (λ= 0.154 nm). The samples were scanned within 2θ of 5-90°at 40 kV and 30 mA with a scanning rate of 5°·min^-1^. The Raman spectra were obtained by a solid-state laser (785 nm) on a Raman spectrometer (France, Horiba JY, Japan). The spectra were recorded over the range from 500 to 3000 cm^−1^. Small-angle x-ray scattering (SAXS) and Wide-angel x-ray scattering (WAXS) measurements were performed using a Xeuss 3.0 SAXS/WAXS system, equipped with an Eiger2R 1M detector, which has a pixel size of 75 μm. The x-ray source used was a copper target (Cu-Kα) operating at 8.05 keV with a wavelength of 1.54189 Å. The experiment was conducted under a vacuum environment (<1 mbar). The sample-to-detector distance was set to 1000 mm and 100 mm to ensure optimal scattering resolution. Analysis of organic elements, we choose to use a conventional combustion method organic element analyzer (Vario EL Cube, Elementar, Germany). The samples were tested in CHNS mode.

**Note S2. Molecular dynamics (MD)** **simulation.**

Classical molecular dynamics (MD) simulations were employed to investigate the interaction between ILs and cellulose by using the GROMACS 2019.4 software package[1].The force-field parameters of PEG and PVA were generated by the Sobtop script based on GAFF force field (J.L. Tian Lu, Sobtop, Version [1.0(dev3)], http://sobereva.com/soft/Sobtop (accessed on Aug. 9, 2022)). Atomic charges were derived from the optimized geometry using the restrained electrostatic potential (RESP) method[2].In addition, the force field parameter of cellulose was described by GLYCAM06 force-field[3].

Each of the cellulose monomer chain contains 10 glucoses repeat unit, and the polymerization degree of PVA and PEG monomer chain is 10 and 20, respectively. All systems are constructed by the Packmol program[4]. The simulation process for all of these systems is carried out according to the following methods: the energy minimization was first performed to remove the bad contact between atoms. Then, the system is under the NVT ensemble lasts for 1 ns with the Berendsen thermostat. After reaching the expected temperature, the subsequent equilibrium is carried out in the NPT ensemble lasts for 1 ns to maintain 1 bar of pressure via the Berendsen pressure coupling algorithm[5]. Finally, for each system, 50 ns MD simulation was performed without any restrictions under the NPT ensemble with a time step of 2 fs. The Lennard−Jones and electrostatic interactions were truncated at cutoff distances of 1.2 nm. The long-ranged electrostatic terms were evaluated using the particle mesh Ewald summation[6]. Bond lengths with hydrogen were constrained with the LINCS algorithm[7]. Periodic boundary conditions were applied in all three dimensions. The trajectories were visualized via the visual molecular dynamics (VMD) 1.9.3 software[8].


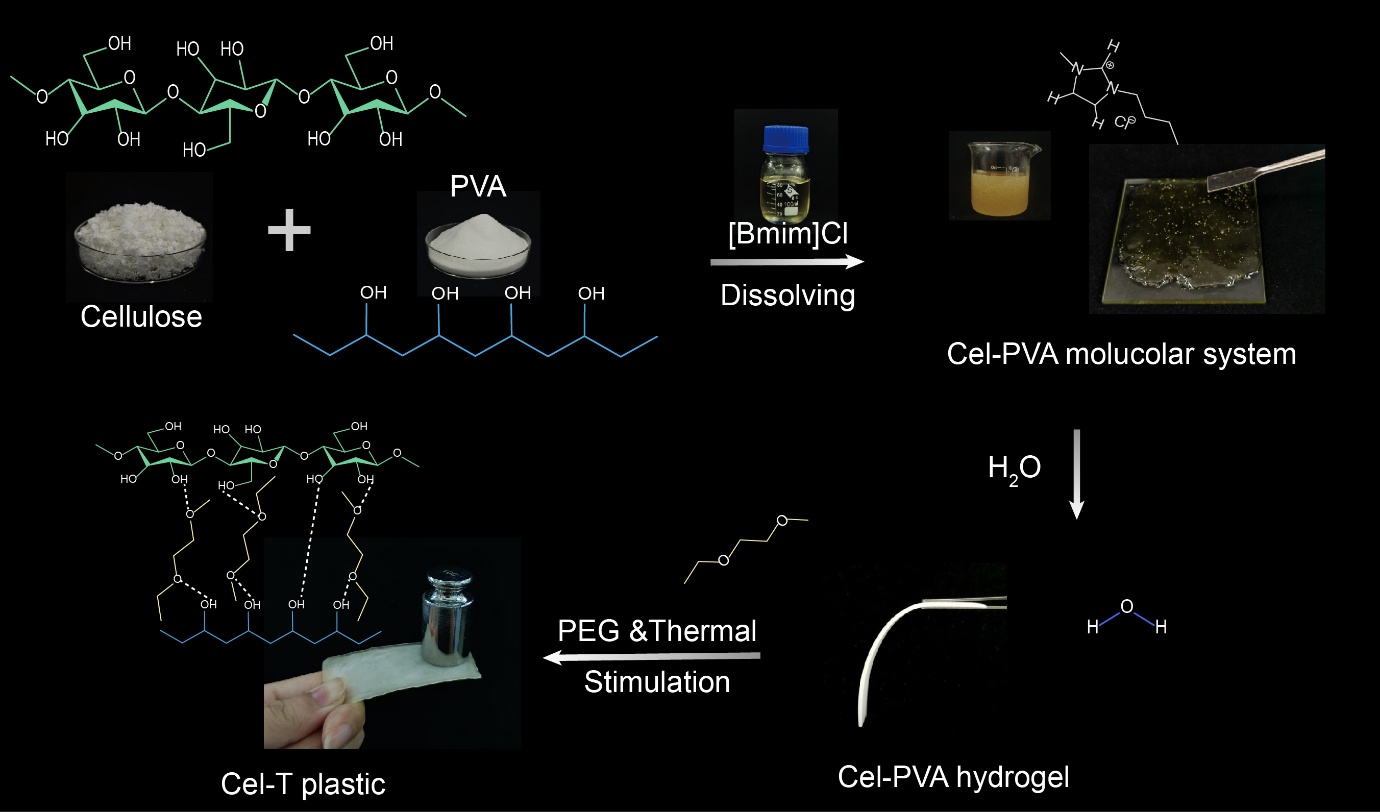


**Figure S1.** Fabrication process of Cel-T plastic.


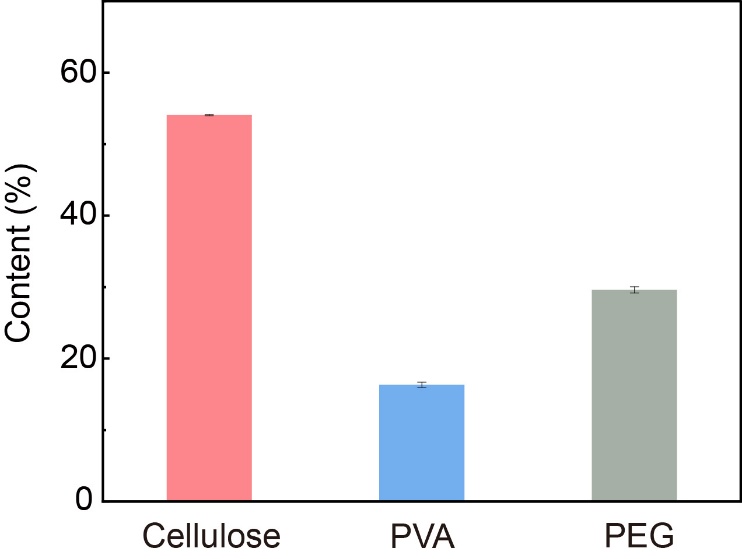


**Figure S2.** The contents of each component in Cel-T plastic.

Quantitative analysis of components: The mass fraction of each component in Cel-T plastic was determined by direct weighing. Specifically, the homogeneous Cel-PVA hydrogel samples with a known dry weight ratio of cellulose to PVA (10:3) were first completely dried and weighed (m_Cel/PVA_). Another gel of the same size was treated with PEG and thermally stimulated to form Cel-T plastic and then weighed (m_Cel-T_). The difference in quality between the two is the mass of the absorbed PEG (m_PEG_). The mass fractions of each component are calculated accordingly.


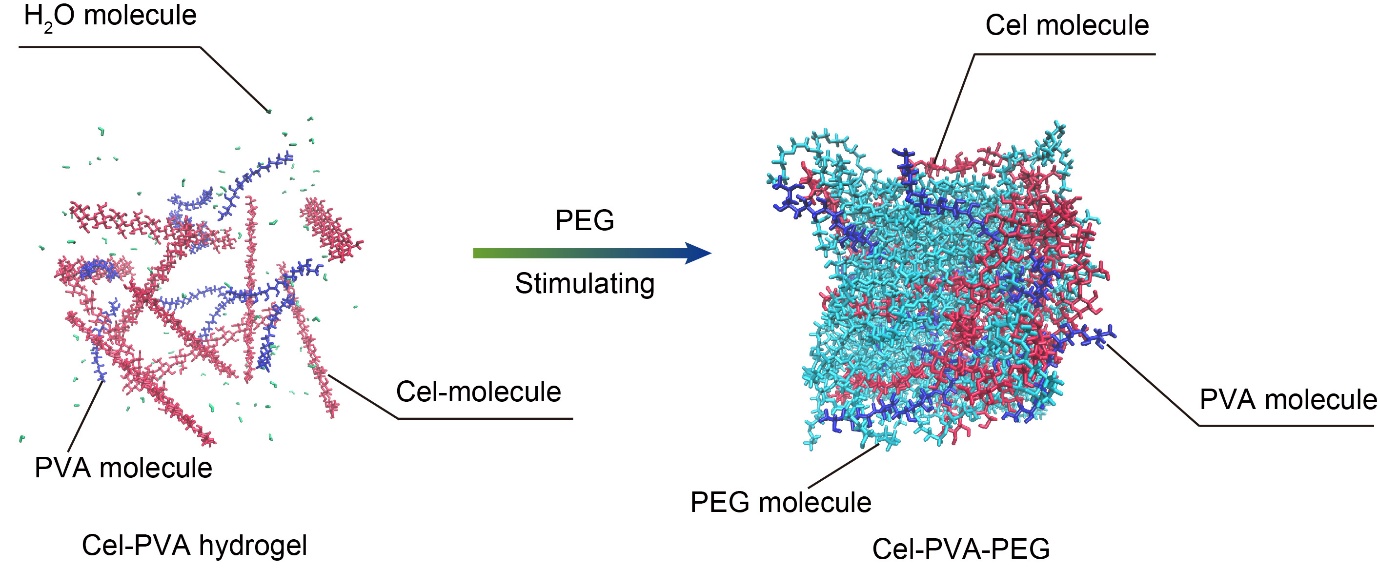


**Figure S3.** The snapshot images of the Cel-PVA hydrogel and the Cel-PVA-PEG system.


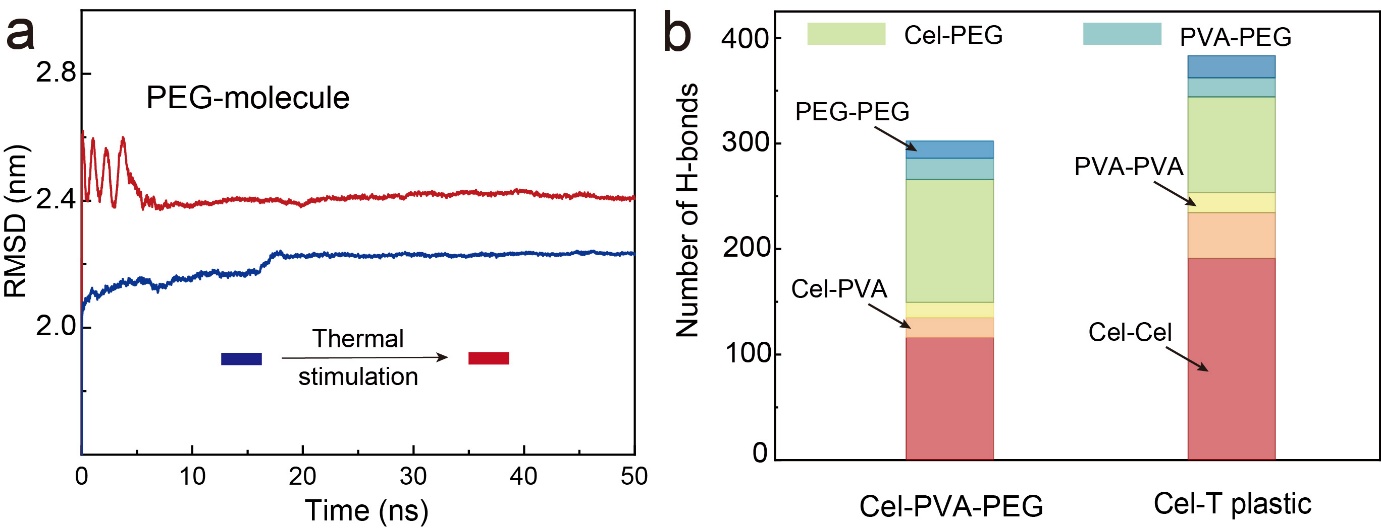


**Figure S4.** MD stimulation. **a,** RMSD of PEG molecule. **b,** Number of H-bonds in the Cel-PVA-PEG system and Cel-T plastic.


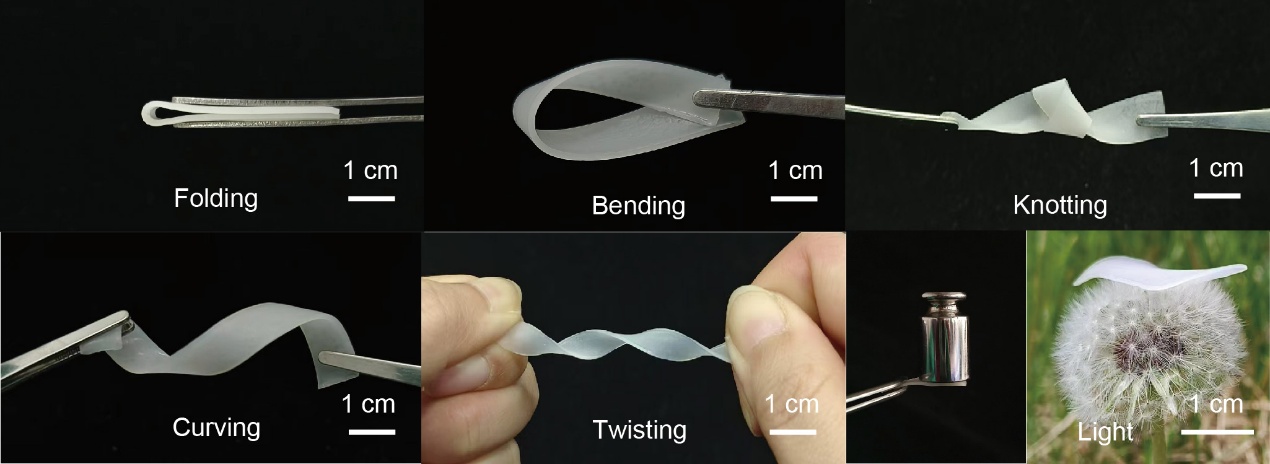


**Figure S5.** Demonstrate the strength and toughness characteristics of Cel-T plastic.

**
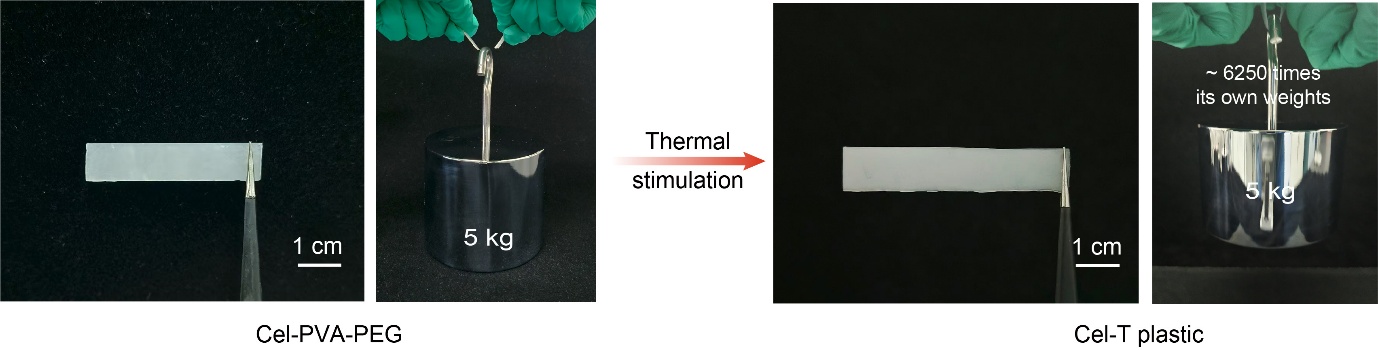
**

**Figure S6.** The photos show two different states, and the Cel-PVA-PEG system under the brittle state cannot lift 5 kg weights, while Cel-T plastic under the robust state can lift 5 kg weights.


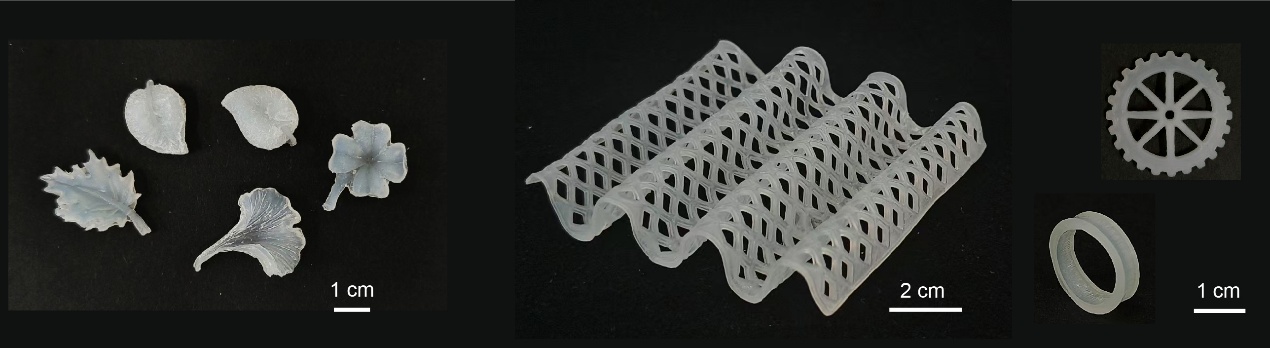


**Figure S7.** Cel-T plastic can be made into leaf shapes, large-sized wavy shapes, gears and rings.


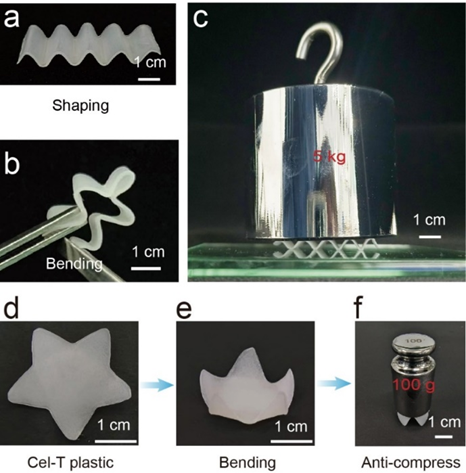


**Figure S8.** Demonstration of the processability and anti-compress ability of Cel-T plastic. **a,** Wave shaping Cel-T plastic. **b,** Bending wave-shaped Cel-T plastic. **c,** The formed Cel-T plastic can withstand the pressure of a 5 kg weight. **d,** The star-shaped Cel-T plastic. **e,** Bending star-shaped Cel-T plastic. **f,** The formed Cel-T plastic can withstand the pressure of a 100 g weight.

**
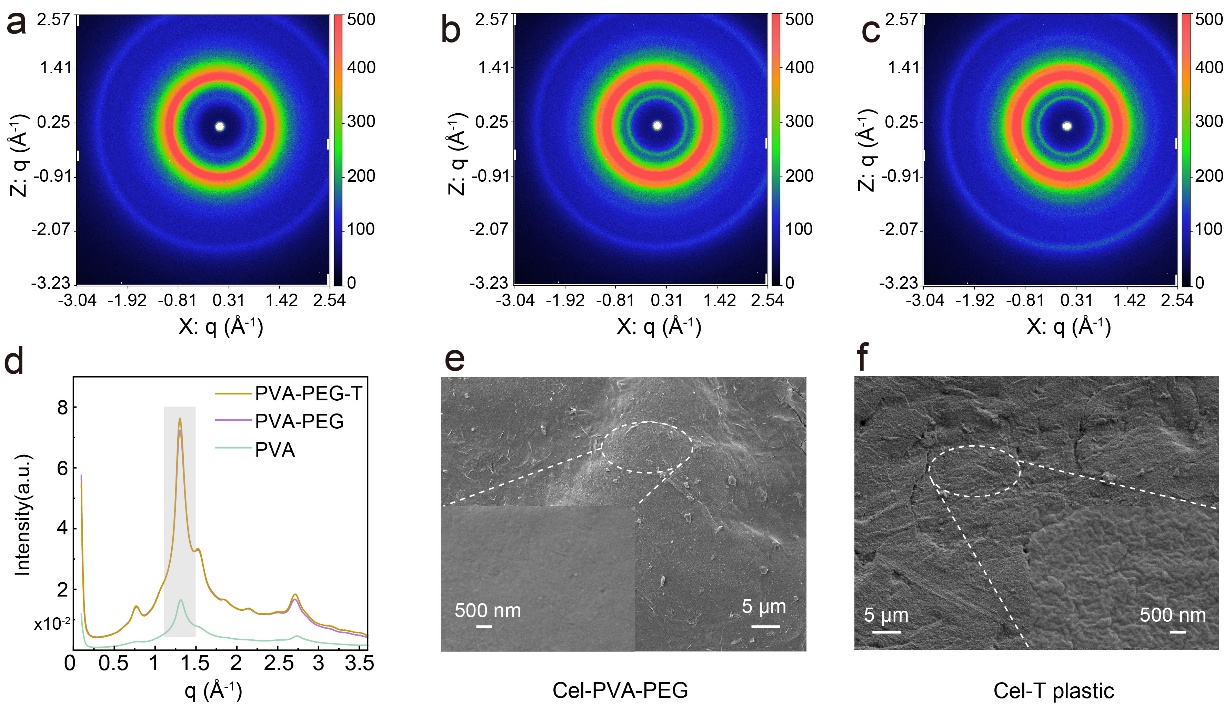
**

**Figure S9.** The WAXS tests and SEM images. **a,** 2D WAXS image of the PVA hydrogel. **b,** 2D WAXS image of the PVA-PEG system. **c,** 2D WAXS image of the PAV-PEG system after thermal stimulation. **d,** WAXS curves of the PVA hydrogel, the PVA-PEG system and PAV-PEG system after thermal stimulation. **e,** SEM images of different magnifications of the Cel-PVA-PEG system. **f,** SEM images of different magnifications of the Cel-T plastic.

The WAXS results are shown in Figure S9. Figures S9a to c respectively corresponds to the 2D WAXS images of the PVA hydrogel, the PVA-PEG system and the PVA-PEG system stimulated by heat. Combined with the 1D WAXS curves (Fig. S9d), it is proved that the peak positions with q values within the range of 1.25 to 1.5 belong to the characteristic peaks of PVA. The peak intensity was higher after thermal stimulation, and the scattering ring corresponding to this position was brighter after thermal stimulation, further verifying that thermal stimulation induces PVA recrystallization.


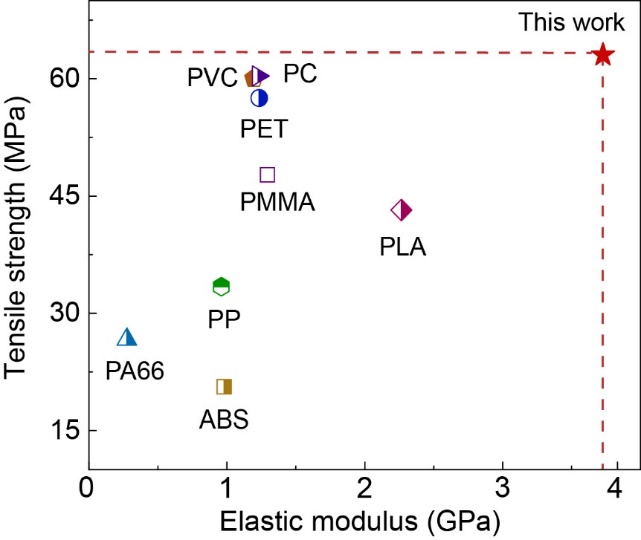


**Figure S10.** The tensile strength and elastic modulus of Cel-T plastic compared with common plastics.


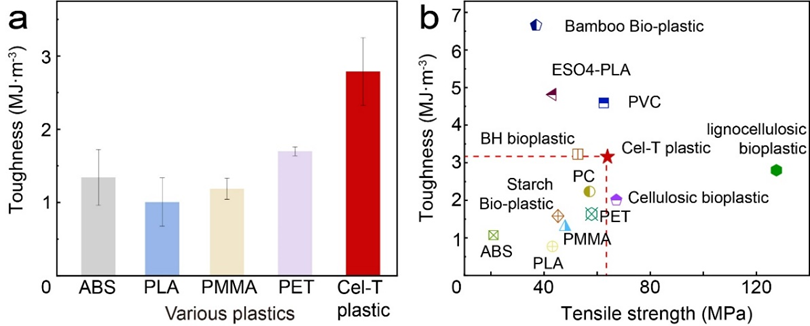


**Figure S11.** The toughness of Cel-T plastic compared with common plastics. **a,** Compared with ABS, PLA, PMMA and PET. **b,** Compared with common plastics, reported modified plastic and bioplastics [9-14].


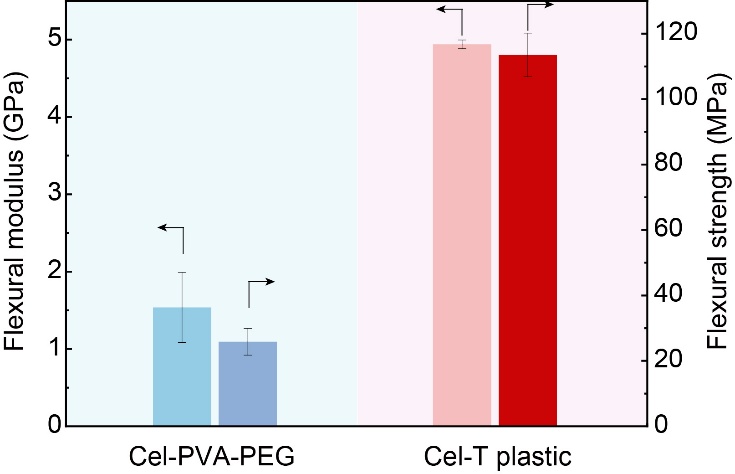


**Figure S12.** The flexural modulus and flexural strength compared Cel-PVA-PEG with Cel-T plastic.


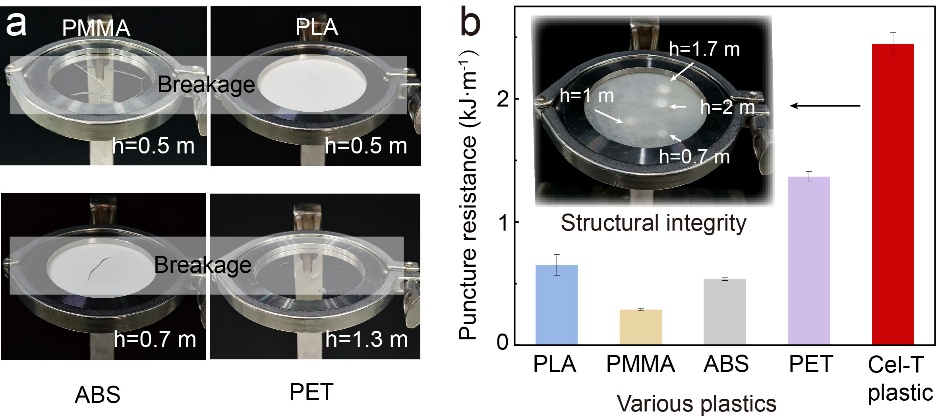


**Figure S13.** Free-fall puncture performance tests. **a,** The breakage states of PMMA, PLA, ABS and PEG after the free-fall puncture performance test. **b,** The puncture marks with different height on Cel-T plastic and the puncture resistance compared Cel-T plastic with PLA, PMMA, ABS and PET.


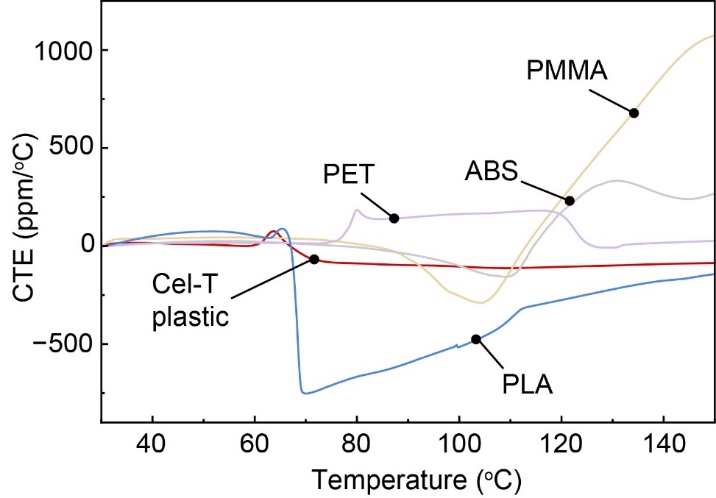


**Figure S14.** The CTE compared Cel-T plastic with common plastics.


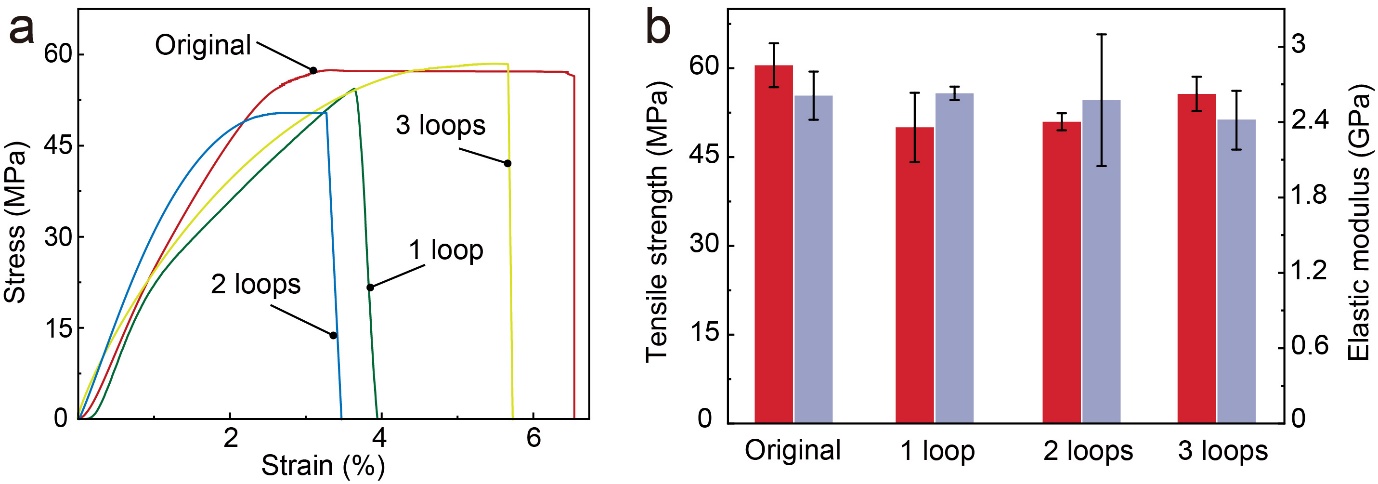


**Figure S15.** Investigating the mechanical performance of Cel-T plastic and recycled Cel-T plastics (prepared from recycled [Bmim]Cl and Cel-T plastic fragments, 1-3 loops). **a,** Tensile stress-strain curves of Cel-T plastic and recycled Cel-T plastics. **b,** Histogram of tensile strength and elastic modulus of Cel-T plastic and recycled Cel-T plastics.


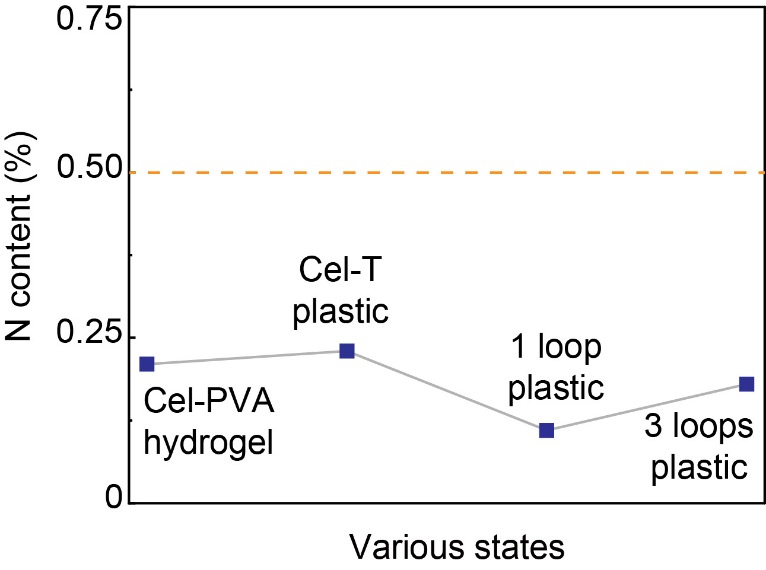


**Figure S16.** The residual content of [Bmim]Cl in the original and recycled Cel-T plastics.


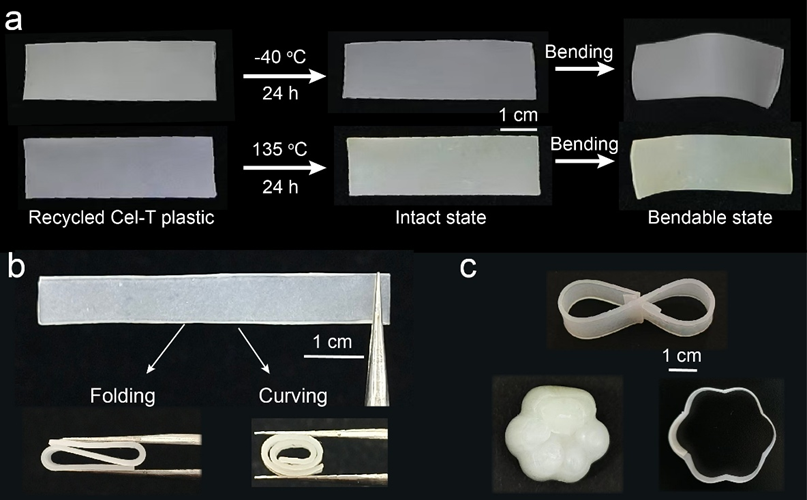


**Figure S17.** The thermal stability and shapeability of recycled Cel-T plastics. **a,** Anti-low temperature and anti-high temperature photos of recycled Cel-T plastic. **b,** Optical images of recycled Cel-T plastic demonstrate excellent toughness and foldability. **c,** Recycled Cel-T plastic exhibits capabilities in weaving, pouring, and molding.

**
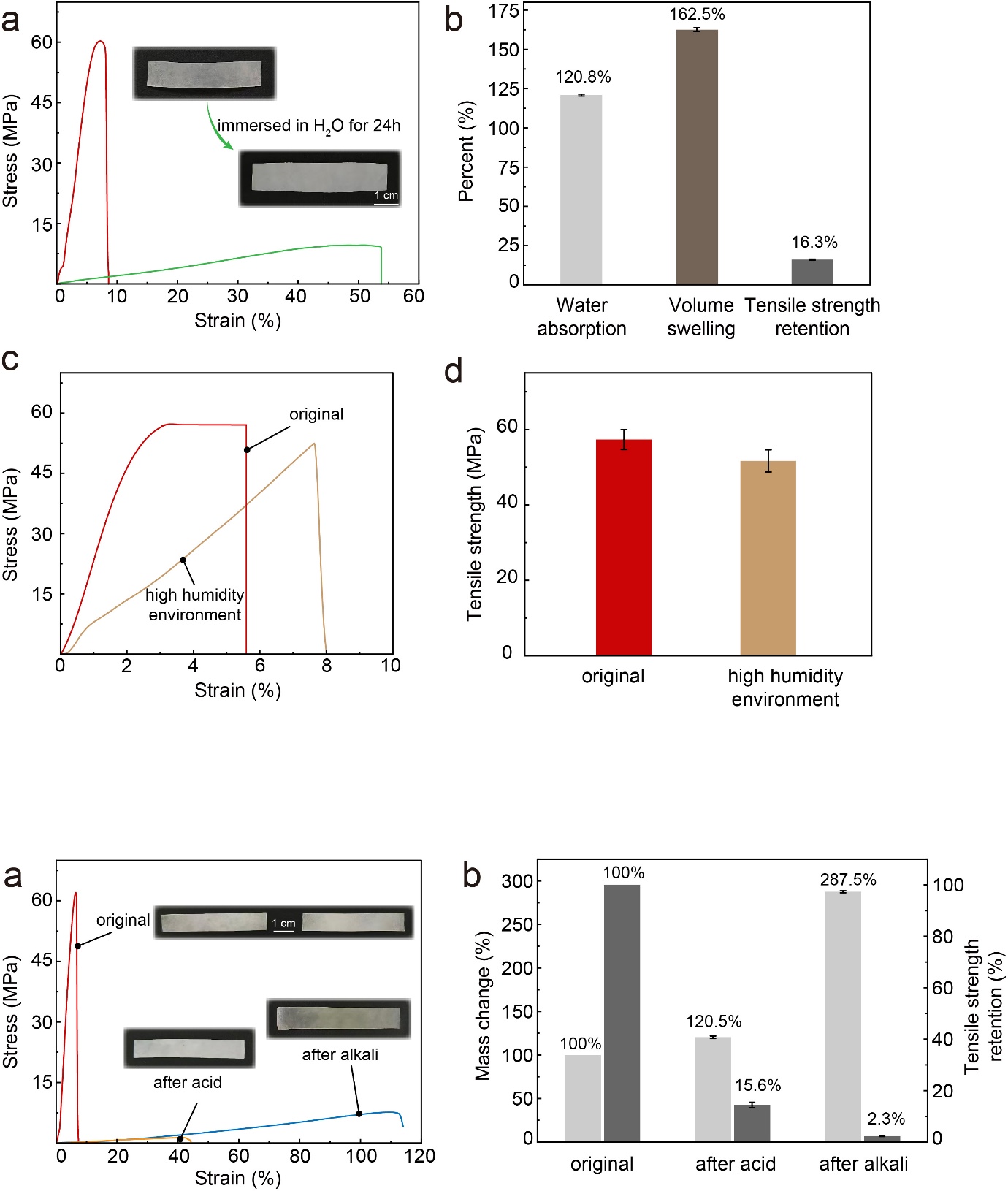
**

**Figure S18.** Investigating the mechanical performance of Cel-T plastic. **a,** Tensile stress-strain curves of original Cel-T plastic and Cel-T plastic after immersed in H_2_O for 24 h. **b,** Histogram of the water absorption, volume swelling and tensile strength retention of Cel-T plastic after immersing in H_2_O for 24 h. **c,** Tensile stress-strain curves of original Cel-T plastic and Cel-T plastic after placed in high humidity environment (90%RH, 35 ℃) for 7 d. **d,** Histogram of tensile strength of Cel-T plastic and after placing in high humidity environment for 7 d.


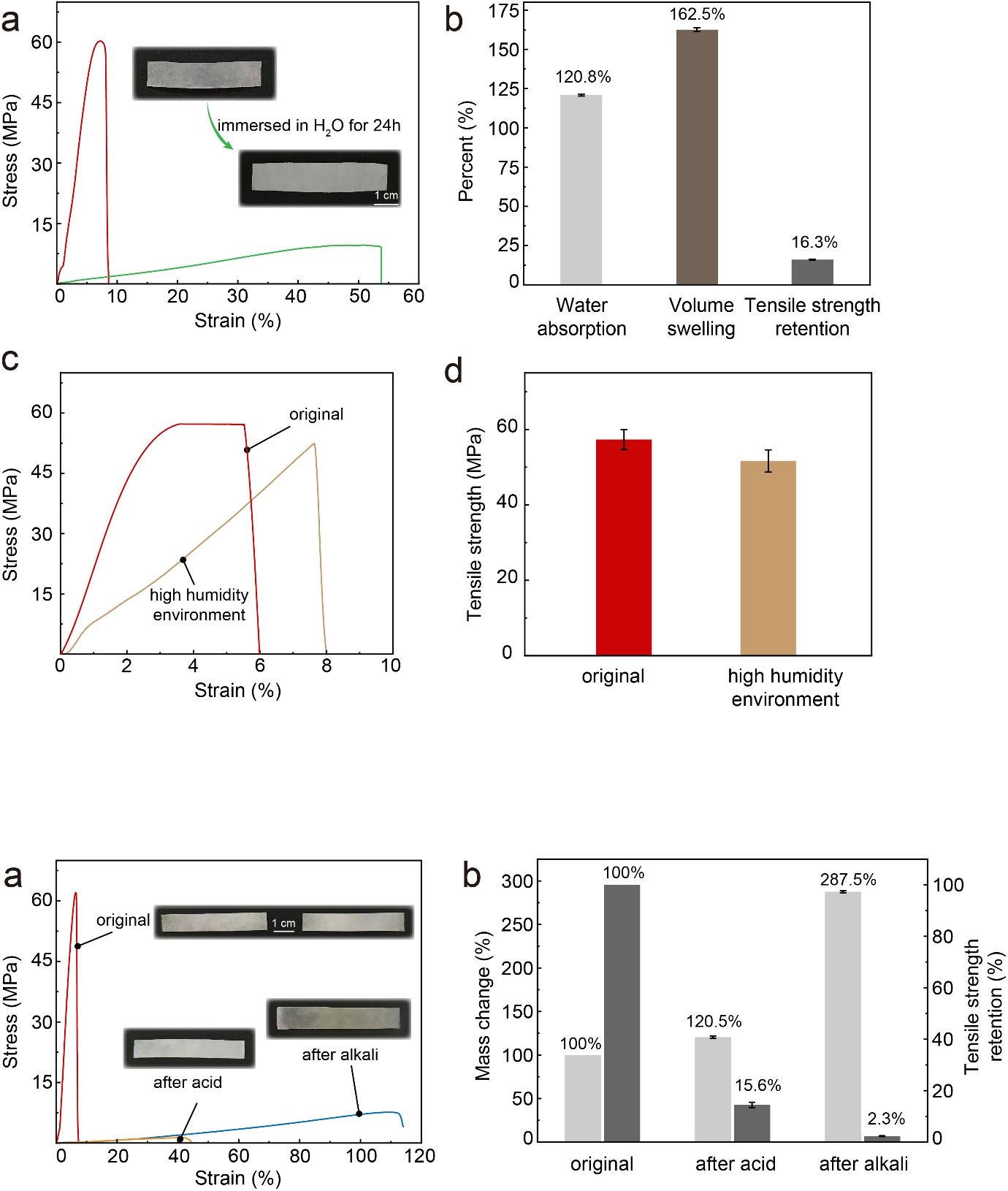


**Figure S19.** The changes of Cel-T plastic after immersion in acidic or alkaline solutions. **a,** The tensile stress-strain curves of Cel-T plastic and Cel-T plastic after soaked in acid (10 wt% CH_3_COOH) and alkali (10 wt% NaOH) solutions for 24 h. **b,** Histogram of mass change and tensile strength retention of Cel-T plastic after soaked in acidic and alkaline solutions.

**Table S1.** Price estimation for Cel-T plastic after 7 cycles. Each cycle results in a loss of 20% of PEG, 17.4% of [Bmim]Cl, and 20% of water.

| **Feedstocks** | **Price**  **($/t)** | **Consumption**  **(t)** | **Cost**  **($)** | **Source** |
| --- | --- | --- | --- | --- |
| Cellulose | 150 | 0.54 | 81.12 | https://www.alibaba.com/product-detail/Hot-Sales-Bleached-Hardwood-Kraft-Pulp_11000024528447.html?spm=a2700.details.you_may_like.3.4b0d4927Kyh5Pr |
| PVA | 1500 | 0.16 | 243.36 | https://www.alibaba.com/product-detail/Polyvinyl-Alcohol-BP17-1788-Polyvinyl-alcohol_1601503907366.html?spm=a2700.galleryofferlist.normal_offer.d_title.4d5d13a0vRYJ91&priceId=f9f62038fe0d4691821d2efb8f25607c |
| PEG | 1500 | 0.35 | 529.02 | https://www.alibaba.com/product-detail/Poly-ethylene-Glycol-Cas-25322-68_1600321194187.html?spm=a2700.galleryofferlist.normal_offer.d_title.1ee913a0vqFbpZ&priceId=d10d52f7522c4bf78d495933741feba5 |
| [Bmim]Cl | 2000 | 1.04 | 2094.08 | https://www.alibaba.com/product-detail/1-Butyl-3-methylimidazolium-Chloride-CAS_1601300481883.html?spm=a2700 |
| H_2_O | 0.3276 | 94.23 | 30.87 | https://scj.shenyang.gov.cn/zwgk/fdzdgknr/gsgg/202503/t20250331_4829895.html |
| Electricity | 0.0697 | 1256 | 87.54 | https://scj.shenyang.gov.cn/zwgk/fdzdgknr/gsgg/202503/t20250331_4829895.html |
| Total cost | 3066 | | | |

**Table S2.** Comparative prices among Cel-T plastic, ABS, PMMA, PLA, PVC, PA66, PP, PC, and PU.

| **Materials** | **Price**  **($/t)** | **Source** |
| --- | --- | --- |
| Cel-T plastic | 3066 | This work |
| ABS | 1500 | https://www.alibaba.com/product-detail/Resin-POLYLAC-PA757-PA-757-High_1600790871882.html?spm=a2700.galleryofferlist.p_offer.d_title.19d24bc4XmNMHB&s=p |
| PMMA | 2100 | https://www.alibaba.com/product-detail/High-Quality-Poly-Methyl-Methacrylate-Sheet_1600161027202.html?spm=a2700.galleryofferlist.normal_offer.d_title.513b13a0dM6ujY&priceId=21696ffde62a45d39acc2c1867ffee00 |
| PLA | 2850 | https://www.alibaba.com/product-detail/ZOVGOV-100-biodegradable-Compostable-Bio-Plant_1601028355454.html?spm=a2700.galleryofferlist.normal_offer.d_title.2731abf5Vm0ZGd |
| PVC | 1550 | https://www.alibaba.com/product-detail/1mm-1-5mm-2mm-Thick-Clear_1601490555784.html?spm=a2700.galleryofferlist.p_offer.d_title.1a2613a0De1M5c&priceId=ed32ea41cae84453be18ae0066802d62 |
| PA66 | 2000 | https://www.alibaba.com/product-detail/Virgin-Nylon-PA66-Plastic-Polyamide-66_1601577977829.html?spm=a2700.galleryofferlist.normal_offer.d_title.4a9613a08DQmbg&priceId=2221fa2be4c6478abc794835f38f920c |
| PP | 1100 | https://www.alibaba.com/product-detail/Pp-Ep300h-PP-3090-PPK8009-PP_1601299875592.html?spm=a2700.galleryofferlist.p_offer.2.da2213a01028h3&priceId=d27739d0d73f4fc8821e5ba309b8cd7a |
| PC | 2250 | https://www.alibaba.com/product-detail/1mm-Solid-clear-Polycarbonate-Pc-Plastic_62095627463.html?spm=a2700.galleryofferlist.normal_offer.d_title.70ed13a07QusTJ&priceId=3af6cb7ed2684bafae97ada75408eafd |
| PU | 2000 | https://www.alibaba.com/product-detail/High-Quality-Customized-Yellow-Polyurethane-PU_1601299645584.html?spm=a2700.galleryofferlist.normal_offer.d_title.77bb13a0LLgqkv&priceId=265dbd9906ba40ff8f6d228f2b2ef0de |

**References**

[1] Abraham MJ, Murtola T, Schulz R, Páll S, Smith J C, Hess B, Lindahl E. GROMACS: High performance molecular simulations through multi-level parallelism from laptops to supercomputers. *SoftwareX.* 2015;119-25.

[2] Wang J, Cieplak P, Kollman P A. How well does a restrained electrostatic potential (RESP) model perform in calculating conformational energies of organic and biological molecules? *J Comput Chem.* 2000;21(12):1049-1074.

[3] Kirschner KN, Yongye AB, Tschampel SM, González‐Outeiriño J, Daniels CR, Foley BL, Woods RJ. GLYCAM06: a generalizable biomolecular force field. Carbohydrates. *J Comput Chem.* 2008;29(4):622-655.

[4] Martínez L, Andrade R, Birgin EG, Martínez JM. PACKMOL: A package for building initial configurations for molecular dynamics simulations. *J Comput Chem.* 2009;30(13):2157-2164.

[5] Berendsen HJ, Postma JPM, Van Gunsteren WF, DiNola A, Haak JR. Molecular dynamics with coupling to an external bath. *J Chem Phys.* 1984;81(8):3684-3690.

[6] Feller SE, MacKerell AD. An improved empirical potential energy function for molecular simulations of phospholipids. *J Phys Chem B.* 2000;104(31):7510-7515.

[7] Hess B, Bekker H, Berendsen HJ, Fraaije JG. LINCS: A linear constraint solver for molecular simulations. *J Comput Chem.* 1997;18(12):1463-1472.

[8] Humphrey W, Dalke A, Schulten K. VMD: visual molecular dynamics. *J Mol Graphics.* 1996;14(1):33-38.

[9] Cui L, Pan M, Zhou Y, Xu H, Ning L, Jia S, Wang X, Su Z. A strong, biodegradable, and closed-loop recyclable bamboo-based plastic substitute enabled by polyimine covalent adaptable networks. *Chem Eng J.* 2023;477146952.

[10] Fang X, Tian N, Hu W, Qing Y, Wang H, Gao X, Qin Y, Sun J. Dynamically Cross-Linking Soybean Oil and Low-Molecular-Weight Polylactic Acid toward Mechanically Robust, Degradable, and Recyclable Supramolecular Plastics. *Adv Funct Mater.* 2022;32(46):2208623.

[11] Qiu Y, Zhang D, Long M, Zhou Z, Gao C, Ma S, Qin J, Chen K, Chen C, Zhao Z. Coassembly of hybrid microscale biomatter for robust, water-processable, and sustainable bioplastics. *Sci Adv.* 2025;11(14):eadr1596.

[12] Xia Q, Chen C, Yao Y, Li J, He S, Zhou Y, Li T, Pan X, Yao Y, Hu L. A strong, biodegradable and recyclable lignocellulosic bioplastic. *NatSustain.* 2021;4(7):627.

[13] Xie D, Zhang R, Song S, Yang S, Yang A, Zhang C, Song Y. Nacre-inspired starch-based bioplastic with excellent mechanical strength and electromagnetic interference shielding. *Carbohydr Polym.* 2024;331121888.

[14] Zhou G, Zhang H, Su Z, Zhang X, Zhou h, Yu L, Chen C, Wang X. A Biodegradable, Waterproof, and Thermally Processable Cellulosic Bioplastic Enabled by Dynamic Covalent Modification. *Adv Mater.* 2023;35(25):2301398.
